# Parcellation of functional subregions using an improved affinity propagation clustering algorithm based on structure‐function coupling

**DOI:** 10.1002/gps3.70056

**Published:** 2026-09-16

**Authors:** Yanling Li, Junbiao He, Shen'ao Li, Hongtao Yi, Fengmei Lu, Jingjing Gao

**Affiliations:** ^1^ School of Electrical Engineering and Electronic Information Xihua University Chengdu Sichuan China; ^2^ School of Life Science and Technology The Clinical Hospital of Chengdu Brain Science Institute, University of Electronic Science and Technology of China Chengdu Sichuan China; ^3^ School of Information and Communication Engineering University of Electronic Science and Technology of China Chengdu Sichuan China

**Keywords:** autism spectrum disorder, cluster analysis

## Abstract

**LEARNING POINTS**

- Integrating structural and functional connectivity via a nonlinear SFC framework improves the biological plausibility and robustness of primary visual cortex (V1) subregion parcellation compared with unimodal methods.
- The dorsal subregion of V1 (Dor‐V1) exhibits selective functional abnormalities in individuals with autism spectrum disorder (ASD), which are significantly correlated with the severity of core social symptoms.
- Dor‐V1 dysfunction may serve as a promising neuroimaging biomarker for patient subtyping and predicting treatment response in ASD, highlighting the clinical translational value of this fine‐grained subregion atlas.

Connectivity‐based parcellation can identify functional subregions, yet prior work often uses single‐modality, linear models that may not fully capture complex structure‐function relationships and typically neglect spatial continuity, limiting anatomical interpretability. To address these limitations, we developed an improved affinity propagation (AP) framework that integrates structural and functional connectivity through nonlinear coupling whilst imposing explicit spatial constraints. This approach generated a stable atlas of the primary visual cortex (V1), which was subsequently applied to cohorts of individuals with autism spectrum disorder (ASD) to provide new insights into visual neurobiology and to identify potential imaging biomarkers for precision diagnosis.

## METHODS

### MRI preprocessing

**FIGURE 1:**
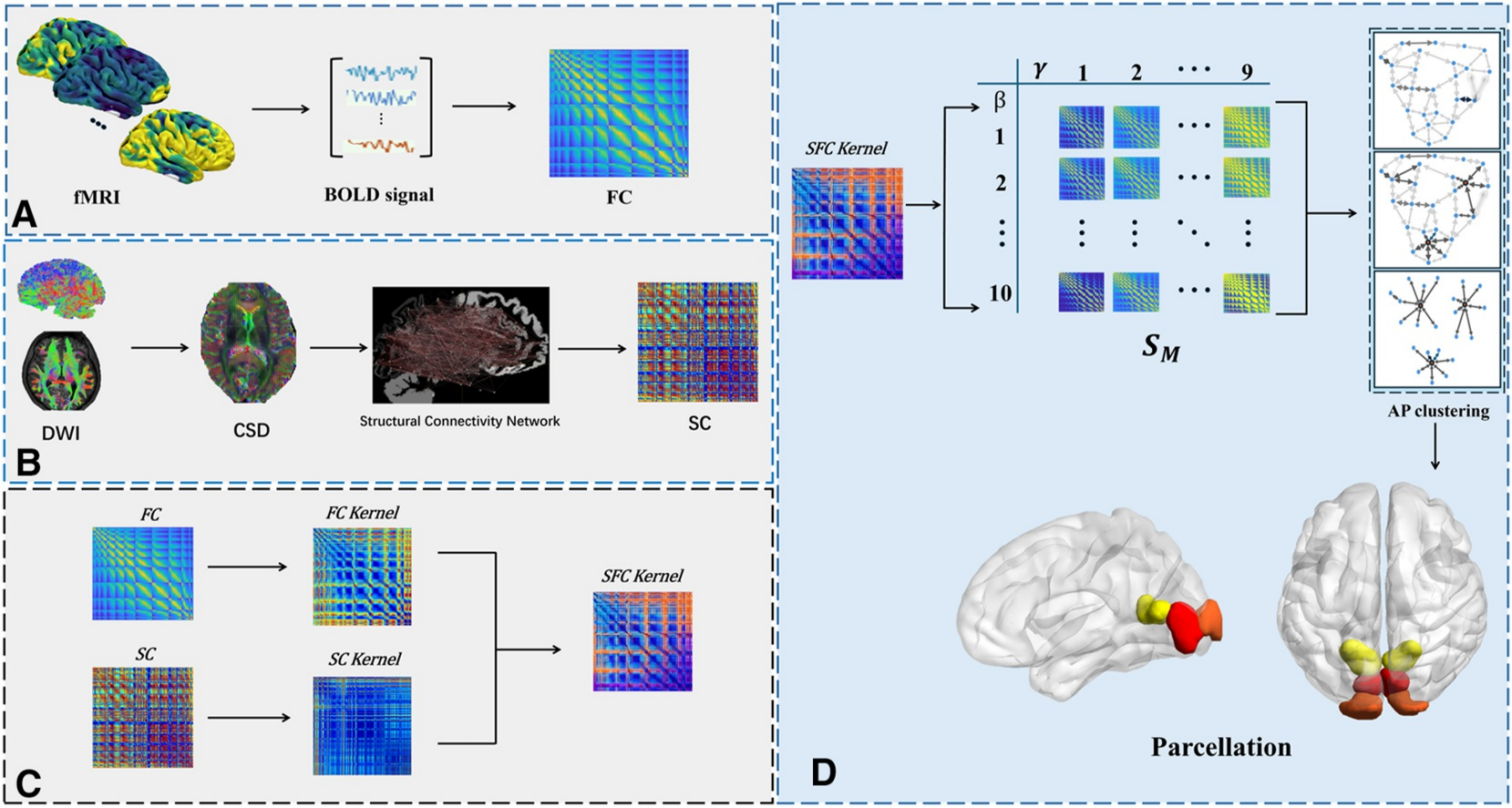
Flowchart of the neighbourhood affinity propagation algorithm with fused connectivity distance constraints. (A) Extraction of BOLD signals from each participant, followed by calculation of the FC matrix for each participant via Pearson correlation. (B) Construction of the SC matrix: DWI data were processed using CSD to obtain a structural connectivity network. (C) Fusion of FC and SC into an SFC matrix via nonlinear kernel space mapping. (D) AP clustering after parameter adjustment and assignment of the SFC matrix. AP, affinity propagation; BOLD, blood oxygen level‐dependent; CSD, constrained spherical deconvolution; DWI, diffusion‐weighted imaging; FC, functional connectivity; fMRI, functional magnetic resonance imaging; SC, structural connectivity; SFC, structure–function coupling.

Resting‐state functional Magnetic Resonance Imaging (rs‐fMRI) data were preprocessed by discarding the initial 10 volumes to allow for signal equilibrium, followed by slice‐timing correction, realignment, normalisation to Montreal Neurological Institute (MNI) space and resampling to 3‐mm isotropic voxels (Figure 1). Nuisance signals (white matter, cerebrospinal fluid, motion parameters and global signal) were regressed out and data were temporally filtered (0.01–0.10 Hz) and spatially smoothed. From the preprocessed data, voxel‐wise indices were computed: amplitude of low‐frequency fluctuations (ALFF), fractional ALFF (fALFF), regional homogeneity (ReHo), degree centrality (DC) and voxel‐mirrored homotopic connectivity (VMHC), followed by standardisation. Diffusion‐weighted imaging (DWI) data underwent correction for eddy‐current distortion and head motion. Fibre orientation distributions were estimated using constrained spherical deconvolution, followed by whole‐brain probabilistic tractography to reconstruct white‐matter pathways. Detailed acquisition parameters and preprocessing settings are provided in the Supporting Information S1: Methods. Participants were excluded if head motion exceeded 2 mm translation or 2° rotation, if mean framewise displacement exceeded 0.5 mm or if a substantial number of volumes were missing or image quality was poor.

### Definition of V1 region

V1 is the first cortical stage of visual processing and is essential for transforming sensory input into higher‐order perception. V1 was defined as Brodmann area 17 using a standard anatomical atlas. Bilateral V1 masks were transformed into MNI space and resampled to match functional image resolution. These masks served as regions of interest (ROIs) for all subsequent connectivity and subregion‐level analyses.

### Construction of functional and structural connectivity matrices

For each voxel within V1, whole‐brain functional connectivity profiles were generated using Pearson correlations between the voxel time series and all other brain voxels. Similarity between voxel‐wise connectivity profiles within V1 was then calculated to construct a functional connectivity (FC) similarity matrix. Structural connectivity (SC) profiles were derived from tractography‐based streamline distributions linking each V1 voxel to the rest of the brain. Similarity between voxel‐wise structural profiles was used to generate an SC similarity matrix. Together, FC and SC matrices provided complementary descriptions of local functional coordination and anatomical wiring patterns. To minimise scanner‐site effects and avoid information leakage, ComBat harmonisation was performed separately for the atlas‐construction and clinical‐validation stages. The detailed mathematical formulation for constructing FC and SC similarity matrices is provided in Supporting Information S1: Section S5.

### Structure‐function coupling matrix

To integrate multimodal information, FC and SC matrices were transformed into kernel space using a nonlinear radial basis function, a strategy informed by recent advances in multimodal fusion.^1^ The transformed matrices were fused to generate a structure‐function coupling (SFC) matrix and subsequently normalised. This representation captures nonlinear interactions between structural and functional connectivity whilst preserving shared organisational patterns. A comprehensive description of the nonlinear kernel space mapping, fusion and normalisation procedures can be found in Supporting Information S1: Section S6.

### Improved clustering framework

We developed an improved AP clustering framework that combined the SFC matrix with spatial distance constraints to enhance anatomical continuity of subregions, aligning with recent methodological advances in multimodal brain subregion frameworks.^2^ Unlike conventional clustering approaches such as k‐means, AP does not require a predefined number of clusters and instead identifies representative exemplars through iterative message passing. Model parameters were optimised using grid search. Candidate solutions were evaluated using silhouette coefficient, Davies–Bouldin index and spatial contiguity. The final subregion solution was selected according to clustering quality, robustness and neuroanatomical plausibility. The complete algorithmic details of the improved AP framework, including parameter optimisation and evaluation criteria, are described in Supporting Information S1: Sections S7 and S8.

### Validation and reproducibility

Atlas stability was assessed using 1000 bootstrap resampling iterations in the training sample. In each iteration, participants were resampled with replacement and clustering was repeated. Dice coefficients between the original and resampled subregions were calculated for each subregion to quantify reproducibility. Further details on the stability analyses, including the impact of different evaluation window widths, are provided in Supporting Information S1: Section S8.

## APPLICATIONS

Autism spectrum disorder (ASD) is a neurodevelopmental condition characterised by social communication deficits and restricted behaviours, frequently accompanied by sensory abnormalities, particularly atypical visual processing.^3^, ^4^ Individuals with ASD commonly exhibit impairments in visual attention, motion perception and visuospatial integration, suggesting that abnormal visual‐system organisation may contribute to core clinical symptoms.^5^, ^6^, ^7^ Early studies suggested links between V1 dysfunction and atypical perception in ASD.^8^ This is corroborated by recent meta‐analytic evidence highlighting altered visual processing and its association with core symptoms in this population.^9^ However, the fine‐scale functional organisation of V1 remains poorly understood.

### Participants

Data were obtained from the Autism Brain Imaging Data Exchange (ABIDE I and II), a publicly available multisite neuroimaging database. After stringent quality control, a total of 479 participants with high‐quality rs‐fMRI data were included, comprising 411 typically developing (TD) controls and 68 individuals with ASD. Of these, 98 participants (including 30 TD individuals) also had complete DWI data, enabling multimodal analyses.

A two‐stage analytical design was adopted to ensure methodological rigour and clear separation between atlas derivation and clinical application:

1. Atlas construction stage: To establish a normative V1 subregion atlas reflecting typical brain organisation, we used only the multimodal (rs‐fMRI and DWI) data from the 30 TD participants. This approach excluded potential disease‐state confounds to derive a stable baseline model of SFC in V1.
2. Clinical validation stage: To independently test the sensitivity and relevance of the derived atlas in ASD, we formed a dedicated validation cohort from the overall sample. This cohort consisted of 68 individuals with ASD and 98 TD controls who were not involved in the atlas construction stage. Only their rs‐fMRI data were used in this stage. The fixed V1 atlas from the atlas construction stage was applied to this independent cohort to extract neural metrics for between‐group comparisons and clinical correlation analyses.

To assess potential site‐related confounding, site‐level age and sex distributions were also evaluated. Detailed demographic characteristics of the cohorts used in each stage are presented in Supporting Information S1: Tables.

### Atlas construction stage

After parameter optimisation, the improved AP clustering framework identified a stable three‐subregion solution for V1. Across different parameter settings, clustering results showed high consistency and bootstrap resampling confirmed strong reproducibility (Dice coefficient > 0.90). Both silhouette coefficient and Davies‐Bouldin index supported the three‐subregion model, indicating robust intrinsic functional organisation of V1. To validate the anatomical correspondence of our parcellation, we computed the spatial overlap between our derived V1 subregions and canonical atlases. Specifically, we quantified the Dice similarity coefficients against the Human Connectome Project (HCP) multimodal atlas^10^ and the Brainnetome atlas,^11^ both mapped to the left hemisphere. Detailed quantitative results are provided in Supporting Information S1: Sections S9.1 and S9.2.

Compared with conventional clustering approaches, including spectral clustering, *k*‐means, hierarchical clustering and standard AP, the proposed method achieved the best overall performance. It produced the highest silhouette coefficient (0.5197) and the lowest Davies‐Bouldin index (0.6936), indicating superior within‐cluster homogeneity and between‐cluster separation. Under simulated Gaussian noise conditions, the proposed method remained stable across all noise levels, demonstrating strong robustness to signal disturbance.

### Clinical validation stage

After applying the atlas to the independent ASD and TD cohort, mean ALFF, fALFF, ReHo, DC, VMHC and ROI‐to‐ROI functional connectivity values were extracted from each V1 subregion. Between‐group differences were tested using analyses of covariance, controlling for age and sex. Multiple comparisons were corrected using false discovery rate (FDR). Within the ASD group, partial correlation analyses were conducted between imaging metrics and Autism Diagnostic Observation Schedule scores, controlling for age and sex. These analyses were used to evaluate the clinical relevance of subregion‐specific abnormalities. Details of the clinical assessment tools and full statistical results are reported in Supporting Information S1: Section S10.

Significant abnormalities were observed mainly in the dorsal V1 subregion (Dor‐V1). Compared with TD controls, individuals with ASD showed reduced ALFF in the left Dor‐V1 and increased DC in the right Dor‐V1 after FDR correction. Effect sizes were medium, with the largest approaching the conventional threshold for a large effect (Cohen's *d* = 0.56–0.77). In addition, reduced interhemispheric connectivity and lower spontaneous activity in Dor‐V1 were significantly associated with greater symptom severity, including social impairment and repetitive behaviours. Complete statistical results, including all *p* values and effect sizes (Cohen's *d*), are reported in Supporting Information S1: Section S10. These findings suggest that dorsal V1 dysfunction may represent a clinically relevant neural marker in ASD.

## DISCUSSION

Using the optimised model, V1 was parcellated into three spatially contiguous subregions: cranial V1 (Cra‐V1), Dor‐V1 and caudal V1 (Cau‐V1). This tripartite organisation suggests that V1 is functionally heterogeneous rather than anatomically uniform. The dorsal subregion may be preferentially involved in visuospatial and motion‐related processing, whereas the caudal subregion may be more closely linked to object‐related visual pathways.

Multimodal SFC outperformed FC alone. The SFC representation showed a higher silhouette coefficient (0.5197 vs. 0.4716) and lower Davies‐Bouldin index (0.6936 vs. 0.7701). A higher silhouette coefficient (closer to 1) indicates better within‐cluster cohesion and between‐cluster separation, whereas a lower Davies‐Bouldin index indicates more compact and well‐separated clusters. Formal definitions of these metrics are provided in Supporting Information S1: Section S8. Functional homogeneity was also higher for SFC than for FC in all three subregions, indicating that integrating structural and functional information improved the biological plausibility of the resulting subregions.

Our findings extend previous connectivity‐based subregion studies by showing that multimodal integration improves robustness and biological interpretability compared with single‐modality methods. Functional connectivity alone is susceptible to physiological noise and inter‐individual variability, whereas structural connectivity provides a relatively stable anatomical scaffold. By combining these complementary modalities, the proposed framework better captured local organisational features of V1 and generated more spatially contiguous subregions. This integrative approach aligns with the methodological frontier in brain network analyses, where the fusion of heterogeneous imaging data has proved valuable for building more accurate and biologically informative models.^12^ Our voxel‐level, region‐specific approach complements recent models of SFC across the whole‐brain network and throughout the lifespan.^9^, ^13^ Structural and functional networks and their coupling can also provide complementary information for characterising brain organisation and neuropsychiatric vulnerability.^14^, ^15^

From a neurobiological perspective, the identified tripartite organisation supports the view that V1 is functionally heterogeneous rather than uniform. In addition, recent meta‐analytic evidence indicates altered local/global visual processing in ASD, characterised by heightened reliance on visual cortical regions.^16^ The dorsal subregion may preferentially participate in visually guided spatial and motion processing, whereas the caudal subregion may be more closely related to ventral‐stream object processing. Such differentiation is consistent with hierarchical models of visual processing and suggests that specialised information processing begins at the earliest cortical stage.

In ASD, abnormalities were primarily localised to the dorsal V1 subregion, where reduced spontaneous activity and altered interhemispheric connectivity correlated with greater symptom severity. This suggests that dorsal V1 dysfunction contributes to core behavioural phenotypes in ASD. As the dorsal visual pathway is essential for processing motion and social cues (e.g., gaze, biological motion), its disruption in V1 may impede bottom‐up transmission of salient visual information, thereby affecting social cognition. This aligns with developmental models in which early alterations in visual processing shape the emergence of core ASD features, including social affect. This result highlights several clinical implications. Subregion‐specific abnormalities offer more sensitive imaging markers than whole‐region measures; for example, the dorsal V1 dysfunction pattern could serve as a potential neuroimaging biomarker for stratifying ASD subtypes based on visual‐processing profiles. Furthermore, this pattern may help investigate developmental trajectories and predict individual responses to interventions targeting sensory integration or social‐cognitive training, ultimately advancing towards precision psychiatry in ASD. Such a multimodal subregion strategy is applicable to other neurodevelopmental and psychiatric disorders with sensory dysfunction. Notably, recent work has demonstrated that accelerated continuous theta‐burst stimulation targeting the left primary motor cortex can improve social communication impairments in children with ASD, supporting the therapeutic potential of non‐invasive neuromodulation. In this context, the identification of clinically relevant visual‐cortical subregions may provide complementary biomarkers for patient stratification, treatment monitoring and the future development of circuit‐based intervention strategies.^17^

The atlas‐based validation further supports the anatomical plausibility of the proposed V1 parcellation. Specifically, the three data‐driven V1 subregions exhibited measurable spatial correspondence with both the HCP multimodal atlas and the Brainnetome atlas, although the overlap was not complete. This observation is expected because both atlases were generated using different parcellation principles, including multimodal cortical features in the HCP atlas and connectional architecture in the Brainnetome atlas, whereas the present study employed a data‐driven functional parcellation strategy. Moderate rather than complete spatial overlap therefore suggests that the proposed subregions preserve the core anatomical organisation of V1 whilst capturing functional heterogeneity that is not reflected in conventional atlas‐defined boundaries.^10^, ^11^

## AUTHOR CONTRIBUTIONS

**Yanling Li:** conceptualization, methodology, software, formal analysis, writing – original draft. **Junbiao He:** data curation, validation, investigation. **Shen'ao Li:** software, visualization, formal analysis. **Hongtao Yi:** resources, data curation. **Fengmei Lu:** project administration, writing – review and editing. **Jingjing Gao:** conceptualization, supervision, funding acquisition, writing – review and editing.

## FUNDING

This work is supported by grants from the National Natural Science Foundation of China (Nos. 62276049 and 61701078) and Sichuan Provincial Science and Technology Support Program (Nos. 2019YJ0193 and 2021YFG0126).

## CONFLICT OF INTEREST STATEMENT

This is the original manuscript and no part of this paper will be considered for publication elsewhere. All authors have approved this manuscript. The authors declare that they have no conflicts of interest.

## ETHICS STATEMENT

The data utilised in this research are sourced from the open‐source ABIDE dataset. Stringent ethical considerations regarding human participants in this study were thoroughly examined and approved by the Institutional Review Board. Before their involvement in the study, written informed consent was diligently obtained from all patients/participants. For instance, studies involving human participants were reviewed and approved by the Ethical Approval Committees at St. James's Hospital/AMNCH (ref: 2010/09/07) and the Linn Dara CAMHS Ethics Committees (ref: 2010/12/07). Written informed consent to participate in this study was provided by the participants' legal guardian/next of kin.

## Supporting information

Supporting Information S1

## Data Availability

The data underlying this article are publicly available in the Autism Brain Imaging Data Exchange (ABIDE) repository.
