# Supplementary material for: Parcellation of functional subregions using an improved affinity propagation clustering algorithm based on structure‐function coupling
Source: Gen Psychiatr. 2026 Sep 16;39(5):e70056. doi: 10.1002/gps3.70056 (PMC13580352; doi:10.1002/gps3.70056)
Supplement: Supplementary file 1 [file GPS3-39-e70056-s001.pdf]

Supplementary material

Contents

Supplementary material..........1

S1. Demographic characteristics of the study cohorts..........3

S1.1 Demographic characteristics of the overall screened resting-state fMRI sample..3

S1.2 Demographic characteristics of the atlas construction cohort..........4

S1.3 Demographic characteristics of the independent clinical validation cohort..........4

S2. MRI acquisition parameters..........4

S3. rs-fMRI preprocessing procedures..........5

S4. Diffusion MRI preprocessing..........5

S5. Construction of FC and SC matrices..........5

S6. Structure-function coupling matrix..........7

S7. Improved affinity propagation clustering..........8

S7.1 Construction of the comprehensive similarity matrix SM..........8

S7.2 Improved AP clustering process..........9

S7.3 Parameter optimisation and optimal solution selection..........10

S8. Clustering evaluation criteria..........10

S9. Spatial overlap (Dice coefficients) between the proposed V1 subregions and atlas-defined V1 regions..........12

S10. Supplementary results..........12

S1. Demographic characteristics of the study cohorts

S1.1 Demographic characteristics of the overall screened resting-state fMRI sample.

Table S1 summarises the final sample of participants with high-quality resting-state functional MRI (rs-fMRI) data that passed all quality control procedures, forming the total pool from which subsequent analytical cohorts were derived.

Table S1

ABIDE I						
Site	Group	Num	Age (mean ± SD)	P value	Sex	P value
Caltech	TD	19	28.9 ± 10.5		15/4	
KKI	TD	24	10.3 ± 1.33		17/7	
Leuven	TD	31	18.0 ± 5.06		26/5	
NYU	ASD	68	15.2 ± 7.03	0.69	58/10	0.0691
	TD	98	15.6 ± 6.22		72/26	
OLIN	TD	10	18.3 ± 3.23		8/2	
PITT	TD	18	19.9 ± 6.46		15/3	
SDSU	TD	20	14.6 ± 1.41		15/5	
STANFORD	TD	14	10.0 ± 1.63		10/4	
UCLA	TD	38	12.9 ± 1.65		32/6	
YALE	TD	23	13.0 ± 2.89		16/7	

ABIDE II						
Site	Group	Num	Age	P value	Sex	P value
NYU_1	TD	28	9.7 ± 3.31		27/1	
SDSU	TD	22	13.1 ± 3.06		20/2	
STANFORD	TD	18	11.0 ± 1.30		16/2	
UCD	TD	14	14.8 ± 1.71		10/4	

UPSM	TD	6	15.9 ± 1.24		4/2	
USM	TD	13	26.0 ± 6.76		11/2	
EMC	TD	15	7.89 ± 0.83		12/3	
Total	ASD	68	15.2 ± 7.03	0.69	58/10	0.0691
	TD	411	14.9 ± 6.62		326/85	
	∑	479			384/95	

Note: For sites contributing only TD participants to the final screened rs-fMRI sample, group-comparison p values are not applicable.

S1.2 Demographic characteristics of the atlas construction cohort (N=30 TD).

This cohort consists of typically developing (TD) participants with complete multimodal (rs-fMRI and DWI) data, used exclusively for deriving the normative V1 parcellation atlas.

Table S2

Site	Group	Num	Age	P value	Sex	P value
NYU	TD	30	10.7 ± 2.61		26/4	
Total	TD	30				

S1.3 Demographic characteristics of the independent clinical validation cohort (N=166).

This independent cohort, comprising individuals not involved in atlas construction, was used to test group differences and clinical correlations in ASD.

Table S3

Site	Group	Num	Age	P value	Sex	P value
NYU	ASD	68	15.2 ± 7.03	0.69	58/10	0.0691
	TD	98	15.6 ± 6.22		72/26	
Total	ASD	68				
	TD	98				
	∑	166				

S2. MRI acquisition parameters

Site-specific MRI acquisition parameters are available from the ABIDE consortium. Only datasets meeting quality-control criteria were included in the present study.

S3. rs-fMRI preprocessing procedures

Resting-state fMRI preprocessing included:

1. Removal of the first 10 volumes
2. Slice-timing correction
3. Head-motion realignment
4. Normalisation to MNI space
5. Resampling to $3 \times 3 \times 3$ mm^3
6. Linear detrending
7. Regression of nuisance covariates (white matter, cerebrospinal fluid, motion parameters and global signal)
8. Temporal band-pass filtering (0.01-0.1 Hz)
9. Spatial smoothing
10. Scrubbing of high-motion time points when necessary

S4. Diffusion MRI preprocessing

Diffusion-weighted imaging data were processed using MRtrix3 and FSL:

1. Eddy-current and motion correction
2. Response function estimation
3. Constrained spherical deconvolution
4. Whole-brain probabilistic tractography
5. Structural connectome construction

Detailed parameters:
- Streamlines: 500 000
- Maximum curvature angle: 45°
- Dynamic step size: 0.5 voxel

S5. Construction of FC and SC matrices

For each voxel in V1:

FC **matrices:**

First, based on the preprocessed rs-fMRI data, the blood oxygen level-dependent (BOLD) time series of each voxel within the bilateral V1 regions of interest (ROIs) was correlated with that of all voxels across the whole brain using Pearson correlation coefficients, thereby generating a whole-brain functional connectivity (FC) map for each V1 voxel. Subsequently, to quantify the similarity

of whole-brain connectivity patterns between any two V1 voxels, we employed the η^2 metric for calculation:

$$\eta^2(a,b) = \frac{\sum_{i=1}^{M}(a_i - m_i)(b_i - m_i)}{\sum_{i=1}^{M}(a_i - \grave{M})(b_i - \grave{M})} \quad (1)$$

Where a_i and b_i represent the functional connectivity strengths of voxel a and voxel b with the i-th voxel across the whole brain, respectively; $\grave{m}_i$ is the average strength of these two voxels at the i-th connection location; and $\grave{M}$ is the overall mean functional connectivity strength of the two voxels across all M connection locations.

By calculating the η^2 value for all pairs of voxels within the V1 region, we constructed the functional connectivity similarity matrix $\mathbf{FC} \in \mathbb{R}^{N \times N}$. This matrix is a symmetric square matrix, where each element $\mathbf{FC}(a,b)$ reflects the similarity of whole-brain functional connectivity patterns between voxel a and voxel b within V1 (as shown in Figure 1a).

SC **matrices:**

Similarly, based on the fibre tractography results, we constructed a whole-brain structural connectivity map for each V1 voxel, where the element values represent the streamline density from that voxel to all other voxels across the whole brain. Using the same η^2 calculation method described above, we quantified the similarity of whole-brain structural connectivity patterns between any two V1 voxels, ultimately constructing the structural connectivity similarity matrix $\mathbf{SC} \in \mathbb{R}^{N \times N}$ (as shown in Figure 1b).

At this stage, we obtained two square matrices, both with dimensions $N \times N$, where N represents the total number of voxels in the V1 region of interest. These two matrices encode the similarity of connectivity patterns between voxels within V1 from functional and structural perspectives, respectively, and will serve as the direct input for subsequent nonlinear fusion.

To avoid information leakage between atlas construction and clinical validation, ComBat harmonisation was performed within each analytical stage without using information from the other stage. In the atlas construction stage, only TD participants were used to derive the normative V1 parcellation atlas. In the clinical validation stage, harmonisation and statistical analyses were conducted within the independent validation cohort, with age and sex controlled in the statistical models. When site harmonisation was required, diagnostic group information was preserved as a biological covariate rather than removed as a batch effect.

- Functional connectivity profiles were computed using Pearson correlations with all brain voxels.
- Structural connectivity profiles were estimated using streamline densities from tractography.
- Voxelwise similarity matrices were constructed using η^2 similarity.

S6. Structure-function coupling matrix

To investigate the intricate coupling relationships between structure and function at the voxel level within brain regions, this study proposes constructing a nonlinear coupling connectivity matrix based on kernel space, termed the Structure-function coupling (SFC) matrix. This method first averages the FC and SC matrices of healthy subjects at the group level to obtain group-averaged FC and SC matrices that represent the population's common patterns. This step aims to derive stable, shared brain connectivity patterns, serving as the foundation for subsequent fusion and parcellation. Subsequently, to capture potential complex, nonlinear interactions between structural connectivity (SC) and functional connectivity (FC) rather than simple linear correlations, we map the group-averaged FC and SC into a high-dimensional Reproducing Kernel Hilbert Space (RKHS) for nonlinear fusion. This process generates a coupling matrix (SFC) that characterises the structure-function interaction patterns. The core advantage of the kernel method lies in its use of a nonlinear mapping function to transform the original connectivity similarities (i.e., the elements in the SC and FC matrices) into a high-dimensional feature space. In this space, originally complex nonlinear relationships may become linearly separable or more easily modelled, thereby more effectively revealing deep-seated associations between multimodal data.

Based on the aforementioned rationale, the construction process of the SFC matrix is divided into the following three steps.

First, kernel matrices are constructed to achieve nonlinear mapping. Both the structural connectivity matrix **SC** and the functional connectivity matrix **FC**, each with dimensions $N \times N$, are transformed using the Gaussian radial basis function (RBF) kernel to map them into a Reproducing Kernel Hilbert Space (RKHS). Here, N represents the number of voxels within the brain region. The Gaussian kernel is selected primarily based on the following two considerations: (1) Universality and smoothness: The Gaussian kernel is a universal kernel function capable of flexibly adapting to different data distributions by adjusting its bandwidth parameter. The feature space it generates is infinite-dimensional and highly smooth, making it suitable for capturing continuous and gradually varying neural connectivity patterns. (2) Neuroscientific plausibility: The strength of connectivity, whether structural or functional, between brain voxels or regions typically decays exponentially or in a Gaussian-like manner with increasing spatial or topological distance. The form of the Gaussian kernel function $K(x,y) = exp(-\gamma||x-y||^2)$ naturally models this property, where similarity smoothly decays with increasing "distance" (here, the difference in the original connectivity patterns). This aligns with the general neurobiological observation of connectivity weight decay. The transformation formula is as follows:

$$K_{SC}\left(i,\ j\right) = e^{(-\gamma_{SC} \cdot SC(i,j)^2)}, \gamma_{SC} = \frac{1}{|SC|} \tag{2}$$

Among them, the adaptive bandwidth parameter γ_{SC} is determined by the reciprocal of the mean absolute value of the elements in the SC matrix. This setting enables the kernel transformation to automatically adjust based on the overall scale of the input data, ensuring numerical stability and adaptability to the intrinsic variance of the data. The functional connectivity kernel matrix K_{FC} is computed in the same manner.

Second, nonlinear fusion of structure and function is achieved in the kernel space. After obtaining

the kernel matrices K_{SC} and K_{FC}, their nonlinear interaction in the kernel space is realised through matrix multiplication, resulting in the initial fusion matrix F:

$$F = K_{\mathrm{SC}} K_{\mathrm{FC}}^{\top} \tag{3}$$

This operation can be interpreted as: first, the functional connectivity patterns are projected into the kernel space via K_{FC}, and then weighted or modulated from the perspective of structural connectivity through K_{SC}. This essentially reflects the nonlinear constraint and shaping relationship of the structural connectivity framework, based on anatomical foundations, on dynamic functional connectivity patterns. To ensure symmetry of the fusion matrix, the following symmetrisation process is applied to F:

$$F \leftarrow \frac{1}{2}(F + F^{\top}) \tag{4}$$

Finally, the fusion matrix is subjected to normalisation. To eliminate bias caused by uneven node degree distribution, a diagonal normalisation method is applied to standardise the fusion matrix:

$$D = \mathrm{diag}\left(\frac{1}{\sqrt{\mathrm{diag}\,(F)}}\right) \tag{5}$$

$$F_{\mathrm{norm}} = D \cdot F \cdot D \tag{6}$$

where D is the inverse square root of the degree matrix. The normalised matrix F_{norm} more robustly represents the relative coupling strength between voxels and can be directly used for subsequent clustering analysis.

The SFC matrix obtained through the above process not only mathematically integrates nonlinear information from structural and functional modalities but also incorporates consideration of fundamental principles such as "structure determines functional boundaries" and "connection strength decays with distance" in the brain. This lays the groundwork for revealing finer and biologically more meaningful organisational patterns of connectivity within brain regions in subsequent analyses (as shown in Figure 1c).

S7. Improved affinity propagation clustering

To achieve fine-grained and spatially continuous parcellation of the visual cortex V1, this study proposes an improved affinity propagation (AP) clustering algorithm that integrates structure-function coupling and spatial distance constraints (referred to as the improved AP clustering method). Building upon the traditional AP clustering framework, this method incorporates dual information sources: structure-function coupling similarity and spatial distance constraints, to construct a comprehensive similarity matrix that drives the clustering process. This results in a contiguous and homogeneous subdivision scheme for brain regions.

S7.1 Construction of the comprehensive similarity matrix S_M

AP clustering takes a similarity matrix as input. To simultaneously leverage both the structure-

function coupling strength and spatial proximity between V1 voxels, we construct the following comprehensive similarity matrix:

$$S_M = \gamma \cdot F_{\text{norm}} + (1-\gamma) \cdot S_d \tag{7}$$

Here, F_{norm} is the normalised structure-function nonlinear coupling matrix, the construction method of which is detailed in Section S6 of this Supplementary Material. This matrix encodes the deep coupling relationships between voxels based on multimodal information. S_d is the spatial distance weighting matrix, used to constrain the spatial continuity of the clustering results, encouraging anatomically neighbouring voxels to be more likely grouped into the same subregion. γ is the fusion parameter ($0 \leq \gamma \leq 1$), which balances the relative weights of multimodal coupling information and spatial constraints in the clustering process.

The spatial distance weighting matrix S_d is computed using a nonlinear transformation of the Euclidean distance via the Sigmoid function:

$$S_d(i,j) = \frac{1}{1 + \exp\left(\alpha \cdot (D_m(i,j) - d_K(i))\right)} \tag{8}$$

Where: D_m is the Euclidean distance matrix between voxels. $d_K(i)$ is the distance from voxel i to its K-th nearest neighbor, with $K = V - \beta$. Here, V is the total number of voxels in the V1 region, and β is a parameter controlling the range of the local neighbourhood. α is the scaling coefficient used to adjust the slope of the sigmoid function, thereby controlling the decay rate of the distance weighting.

In this study, the parameter β was traversed within the range $[10, 100]$ with a step size of 10, and the parameter γ was traversed within the range $[0, 0.9]$ with a step size of 0.1, to systematically explore the impact of different parameter combinations on the clustering results. To provide a more intuitive understanding of the principle of distance weighting, a schematic diagram S2 of the algorithm is included in the supplementary materials.

S7.2 Improved AP clustering process

Using the constructed comprehensive similarity matrix $\mathbf{S}_M$ as input, the following AP clustering process is executed:

a. Message Passing Iteration

The AP algorithm identifies cluster centres by iteratively updating the **responsibility matrix R** and the **availability matrix A**.

Responsibility matrix R: Represents the cumulative evidence that data point k is suitable as the cluster centre for data point i.

$$R(i,k) \leftarrow S_M(i,k) - \max_{k' \neq k} \{A(i,k') + S_M(i,k')\} \tag{9}$$

Availability matrix A: Represents the cumulative evidence that data point i selects data point k as its cluster centre.

$$A(i,k) \leftarrow \min \left\{ 0, R(k,k) + \sum_{i' \notin \{i,k\}} \max\ (0, R(i',k)) \right\} \quad (10)$$

Its diagonal elements are updated as:

$$A(k,k) \leftarrow \sum_{i' \neq k} \max\ (0, R(\ i',k)) \quad (11)$$

b.

Damped Update and Convergence Judgment

To prevent oscillation during iteration, a damping coefficient λ_{damp} (typically set between 0.1 and 0.9) is introduced for smooth updates:

$$R_{\text{new}} \leftarrow (1 - \lambda_{\text{damp}}) \cdot R_{\text{new}} + \lambda_{\text{damp}} \cdot R_{\text{old}} \quad (12)$$

$$A_{\text{new}} \leftarrow (1 - \lambda_{\text{damp}}) \cdot A_{\text{new}} + \lambda_{\text{damp}} \cdot A_{\text{old}} \quad (13)$$

When the matrix $\mathbf{R} + \mathbf{A}$ remains unchanged over multiple consecutive iterations, the algorithm converges. Ultimately, points satisfying $\mathbf{R}(i,i) + \mathbf{A}(i,i) > 0$ are selected as cluster centres, while the remaining points are assigned to their most similar cluster centre.

S7.3 Parameter optimisation and optimal solution selection

To obtain an optimal and stable V1 subregion scheme, we adopted a systematic parameter optimisation strategy:

(1) Parameter grid search: all candidate combinations of the fusion and spatial-constraint parameters were evaluated. (2) Clustering evaluation: for each parameter set, AP clustering was performed and evaluated using the silhouette coefficient, Davies-Bouldin index and spatial contiguity index. (3) Stability testing: the stability of evaluation metrics was monitored across iterations, and the parameter combination with the best balance of clustering quality, spatial continuity and robustness was selected as the final V1 parcellation solution. This optimised AP framework was designed to generate a fine-grained V1 parcellation scheme with clear neurobiological relevance, high spatial continuity and robustness to data noise. Figure S1 illustrates the parameter traversal, similarity-matrix update and evaluation procedures. A simplified example using a small similarity matrix is also provided to demonstrate the computational steps of AP clustering.

S8. Clustering evaluation criteria

To assess the performance of the clustering algorithm, this study employs multiple evaluation metrics. First, the silhouette coefficient (SH) is used to measure the clustering effectiveness, defined as follows:

$$SH(i) = \frac{b(i) - a(i)}{max\{a(i), b(i)\}} \quad (14)$$

Where a represents the intra-cluster similarity, defined as the average distance between data

point i and all other points within the same cluster; and b represents the inter-cluster dissimilarity, defined as the minimum average distance between point i and all points in any other cluster. The formula for calculating the average silhouette coefficient is:

$$AS = \frac{1}{N}\sum_{i=1}^{N} SH(i)(15)$$

Where N is the total number of data points.

Secondly, the Davies-Bouldin Index (DBI) is also an important metric for evaluating clustering effectiveness, defined as:

$$DBI(K) = \frac{1}{K}\sum_{i=1}^{K} \max_{i \neq j} \left\{ \frac{S_i + S_j}{d(x_i, x_j)} \right\}(16)$$

$$S_i = \frac{1}{n_i}\sum_{j=1}^{n_i} d(x_i, x_j)(17)$$

Where S_i is the average distance within cluster i, and $d(\dot{x}_i, \dot{x}_j)$ is the distance between the centroids of cluster i and cluster j. The DBI ranges from $[0, +\infty)$, with smaller values indicating better clustering performance.

Additionally, the Spatial Contiguity (SC) metric is used to evaluate the anatomical coherence of the clustering results, defined as:

$$SC_i = f\left(subregion_{matrix},\ \text{ClusterConnectivityCriterion}\right)(18)$$

$$mean_{sc} = \frac{1}{N}\Sigma_{i=1}^{N} SC_i(19)$$

Where SC_i represents the spatial contiguity of the i-th cluster, f is a computational function based on the subregion matrix and connectivity criteria, and N is the total number of clusters. A larger value of mean_{sc} indicates better anatomical coherence of the clustering results.

This study also investigated the impact of different evaluation window widths (25, 50, 75, 100) on clustering stability. The consistency of clustering results across different windows was compared using the Dice coefficient. The formula for calculating the Dice coefficient is:

$$\text{Dice} = \frac{2 \times |X \cap Y|}{|X| + |Y|}(20)$$

Where X and Y represent the cluster labels from two different clustering results, respectively.

S9. Spatial overlap (Dice coefficients) between the proposed V1 subregions and atlas-defined V1 regions.

To evaluate the anatomical validity of the proposed V1 subregions, spatial overlap analyses were performed using the Glasser atlas and the Brainnetome atlas. Dice coefficients were calculated between each V1 subregion and the corresponding atlas-defined V1 region. For the Glasser atlas, the Dice coefficients were 0.3618 for Cra-V1, 0.2544 for Dor-V1 and 0.1410 for Cau-V1. For the Brainnetome atlas, we identified the best-matched visual cortical ROIs for each proposed V1 subregion and reported the corresponding Dice coefficients in Table S5.

Table S4 Spatial overlap between proposed V1 subregions and the HCP multimodal atlas

V1 Subregion	Voxels(n)	Glasser V1 (Dice)
Cra-V1	182	0.3618
Dor-V1	211	0.2544
Cau-V1	175	0.1410

Note: All analyses were restricted to the left hemisphere. Dice coefficients were calculated to quantify spatial overlap between each proposed V1 subregion and the atlas-defined left V1 region

Table S5 Spatial overlap between proposed V1 subregions and Brainnetome visual cortical regions

Proposed Subregion	Best-matched Brainnetome ROI	Dice coefficient
Cra-V1	BN_190	0.2735
Cra-V1	BN_192	0.4237
Cra-V1	BN_194	0.1564
Dor-V1	BN_190	0.0737
Dor-V1	BN_194	0.2222
Cau-V1	BN_192	0.1435

S10. Supplementary results

To systematically validate the performance of our proposed distance-constrained improved Affinity Propagation (AP) clustering algorithm for fine-grained brain parcellation, we first comprehensively evaluated the robustness of its key parameters and its overall performance relative to mainstream clustering methods. For determining the optimal number of clusters, we adopted a strategy based on identifying smooth regions of gradient changes in silhouette coefficient (SH) and Davies-Bouldin index (DBI) values, this provided a basis for selecting the optimal preference parameter (p) value. To identify such smooth variation intervals, we tested four window widths: 25, 50, 75 and 100 (Figure S1-A), and assessed clustering results using the Dice coefficient for each width. Results showed that a window width of 50 achieved an ideal balance between result stability

and computational efficiency. Specifically, a width of 50 means the sliding window included 50 iterations per evaluation, used to calculate the average SH and DBI values for smooth variation. Notably, the Dice coefficient remained high regardless of window width, indicating low sensitivity of clustering results to this parameter. Thus, we set the window width to 50 as the foundation for subsequent clustering analyses.

Building on this, we introduced two key parameters, γ (nonlinear kernel weight) and β (distance weight), into the improved Affinity Propagation (AP) clustering algorithm to effectively suppress noise and enhance spatial continuity of the data. Specifically, we performed clustering analysis on the coupled structure-function coupling (SFC) matrix and obtained 90 sets of clustering results by varying combinations of γ and β (Figure S1-B). When evaluating these results, we focused on two metrics: the silhouette coefficient (SH; Figure S1-C, left) and the Davies-Bouldin index (DBI; Figure S1-C, right), and identified two optimal parameter sets: $\gamma=0.5$ and $\beta=100$ for SH alone, and $\gamma=0.9$ and $\beta=100$ for DBI alone (Figure S1-C). Although the DBI-based parameter set offered advantages in clustering compactness, it exhibited poorer stability and spatial continuity, suggesting that prioritising compactness alone may fail to capture biologically meaningful patterns in connectivity analysis. In contrast, the SH-based parameter set not only demonstrated superior stability but also stronger spatial continuity. Further spatial continuity assessment (Figure S1-D) quantified these differences: the spatial continuity (SC) value for the DBI-optimal set was 0.9668, versus 0.9916 for the SH-optimal set. Higher SC values indicate more spatially contiguous functional modules, aligning with the actual anatomical structure of brain functional regions, highlighting the critical role of spatial proximity in brain organisation and enhancing both result credibility and biological interpretability. Integrating these findings, we designated the SH-based result as the optimal solution and the DBI-based result as the suboptimal solution. Notably, both metrics consistently supported a 3-cluster solution, confirming that the primary visual cortex (V1) harbours three stable, intrinsic functional modules in its connectivity architecture. This not only validates the robustness of our method across parameter variations but also reveals fundamental organisational principles of brain connectivity.

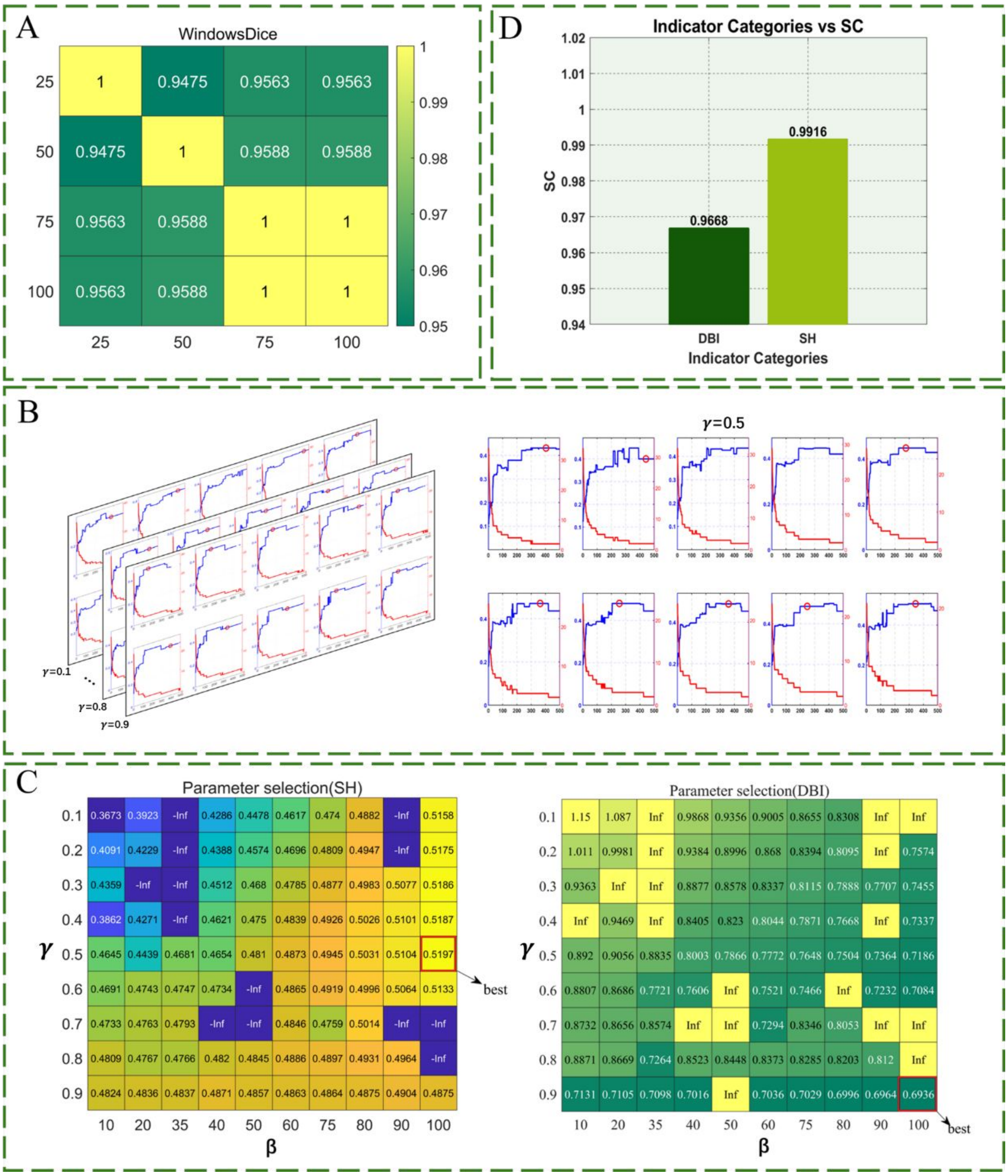

Figure S1. Optimal parameter selection for AP Clustering based on a multi-criteria decision framework. A. Window Length Sensitivity Analysis; B. Parameter Space Exploration and Preliminary Evaluation; C. Parameter Screening Based on Internal Metrics (DBI/SH); D. Final Decision with the Introduction of the Spatial Constraint Metric (SC).

Panel A. The Dice coefficient was calculated for clustering labels under different window lengths (1, 2, 3 and 4 corresponding to stable interval ranges of 25, 50, 75 and 100, respectively). The Dice coefficient ranges from 0 to 1, with higher values indicating better performance. The results in the table show that the clustering results are almost unaffected by the window length.

Panel B. Results of AP clustering under 90 different parameter combinations. For each value of the fusion parameter γ, there are ten sets of results corresponding to different values of the local neighbourhood parameter β, with β increasing from left to right and top to bottom. In each sub-plot, the blue and red curves represent the average Silhouette Coefficient (SH) and the number of clusters, respectively. The red circles mark key evaluation points for each parameter combination. The optimal parameter combination is identified as $\gamma = 0.5$, $\beta = 100$.

Panel C. Illustration of determining optimal parameters using the Davies-Bouldin Index (DBI) and the Silhouette Coefficient (SH). The

bold boxes indicate the optimal parameter sets for each metric. "Inf" values indicate that the condition of a window width of 50 is not satisfied for that parameter set.

Panel D. Both DBI and SH are used to evaluate the clustering results. One clustering result is determined as optimal based on the DBI coefficient, and another is determined as optimal based on the SH coefficient. To resolve the conflict between the two metrics and identify the best overall clustering result, the Spatial Contiguity (SC) coefficient is introduced. The SC values are compared for the results selected by SH and DBI. The clustering result that satisfies the SH-optimal condition while achieving the highest SC value is considered the best overall, confirming SH as the leading metric for this dataset.

To further validate the reliability of our clustering approach, after determining the algorithm's optimal configuration, we compared it with traditional clustering methods along two dimensions, comprehensive performance and noise robustness. Specifically, we conducted a horizontal comparison of results from the original Affinity Propagation (AP) algorithm, spectral clustering, k-means clustering and hierarchical clustering (Figure S2-A, B), followed by a one-way analysis of variance (ANOVA) and Tukey HSD post hoc tests to compare silhouette coefficient (SH) and Davies-Bouldin index (DBI) values across methods. The results showed that the improved AP method yielded a significantly higher SH value (M = 0.5197, SD = 0.011) than spectral clustering (M = 0.3812, SD = 0.015), k-means clustering (M = 0.4024, SD = 0.012) and hierarchical clustering (M = 0.3572, SD = 0.016), all $p < 0.05$. This indicates that the improved AP method is more effective at identifying clusters with high internal similarity and low inter-cluster similarity in the feature space. In addition, the improved AP method produced a significantly lower DBI value (M = 0.6936, SD = 0.016) than spectral clustering (M = 0.9628, SD = 0.020), k-means clustering (M = 0.9268, SD = 0.021) and hierarchical clustering (M = 0.9596, SD = 0.012).

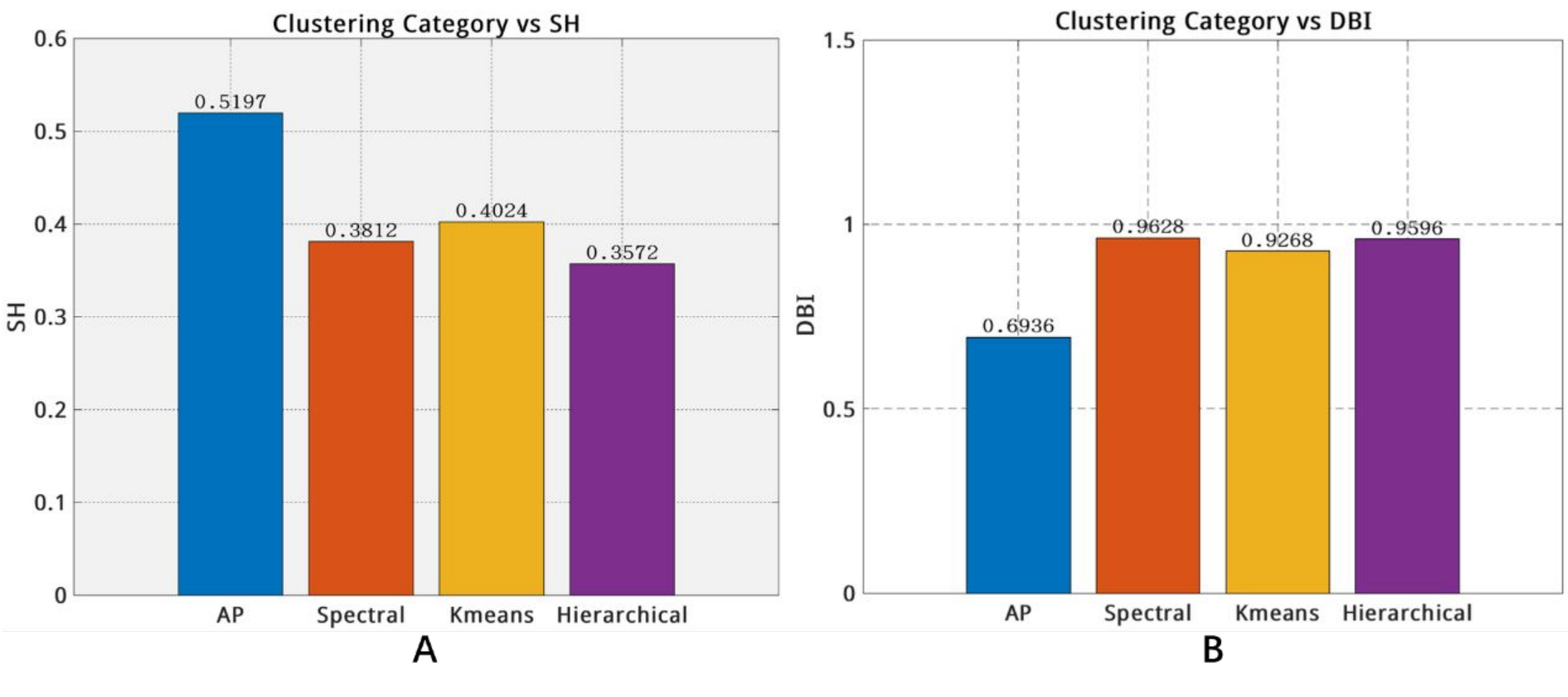

Figure S2 compares the clustering performance among different algorithms. The bar chart displays the average silhouette coefficient (SH, left) and Davies-Bouldin index (DBI, right) for four clustering methods: Affinity Propagation (AP), spectral clustering, k-means and hierarchical clustering. A higher SH value indicates better intra-cluster compactness and inter-cluster separation, whereas a lower DBI value signifies superior clustering quality. Among all methods, the AP-based approach proposed in this study achieved the highest SH value (0.5197) and the lowest DBI value (0.6936), demonstrating the optimal balance between intra-cluster cohesion and inter-cluster separation.

In real-world brain parcellation, BOLD signals are influenced by multiple factors (e.g. motion

artefacts, instrument noise), and noise simulation helps better evaluate the robustness of clustering algorithms in real-world settings. To reveal the stability and adaptability of clustering algorithms, we performed a comparative analysis of several clustering methods with added noise: before calculating Pearson correlation, we added eight levels of Gaussian white noise (0.5, 1, 1.5, 2, 2.5, 3, 3.5, 4) to the BOLD time series to simulate varying degrees of noise interference.

In the noise experiment, designed to assess the partitioning performance of four clustering methods (AP, spectral clustering, k-means and hierarchical clustering) on connectivity data under different noise levels, we set noise parameters (from 0.5 to 4) and calculated the Davies-Bouldin index (DBI) and silhouette coefficient (SH) for each method under each noise condition (Figure S3). The improved AP clustering maintained relatively stable DBI values across all noise levels, ranging from 0.692 to 0.693, indicating good clustering uniformity and separation. Its SH values also showed minor fluctuations (0.49 to 0.491) across noise conditions, reflecting stable cluster compactness. Thus, the improved AP clustering was robust to noise and effectively handled connectivity data under varying noise levels.

Spectral clustering performed worst in terms of DBI: at a noise level of 4, its DBI value reached 0.856 (the highest among all methods), indicating poor inter-cluster separation and compactness under high noise. Its SH values (0.424-0.428) were lower than those of the improved AP, suggesting poorer cluster compactness and lower matching between data points and their clusters. K-means clustering had stable DBI values (0.823-0.830) across noise conditions, showing good separation, but its SH values fluctuated, reaching 0.447 (the highest among methods) at a noise level of 4, indicating higher compactness and consistency at that noise level. However, k-means performed well only at low noise; its stability and compactness slightly decreased as noise increased. Hierarchical clustering had the lowest DBI (0.822) at a noise level of 0.5, but its DBI gradually increased to 0.856 with higher noise, meaning it maintained good separation at high noise. Yet, its SH values fluctuated significantly (dropping to 0.396 at a noise level of 1.5), indicating poor compactness under certain noise conditions. Although its SH values recovered slightly at high noise, they remained lower than those of other methods, reflecting poor cluster consistency.

These results further demonstrate that the improved AP clustering method generates more compact and well-separated clusters, significantly reducing fuzziness and overlap between clusters, an advantage that is particularly prominent when processing high-dimensional, complex data.

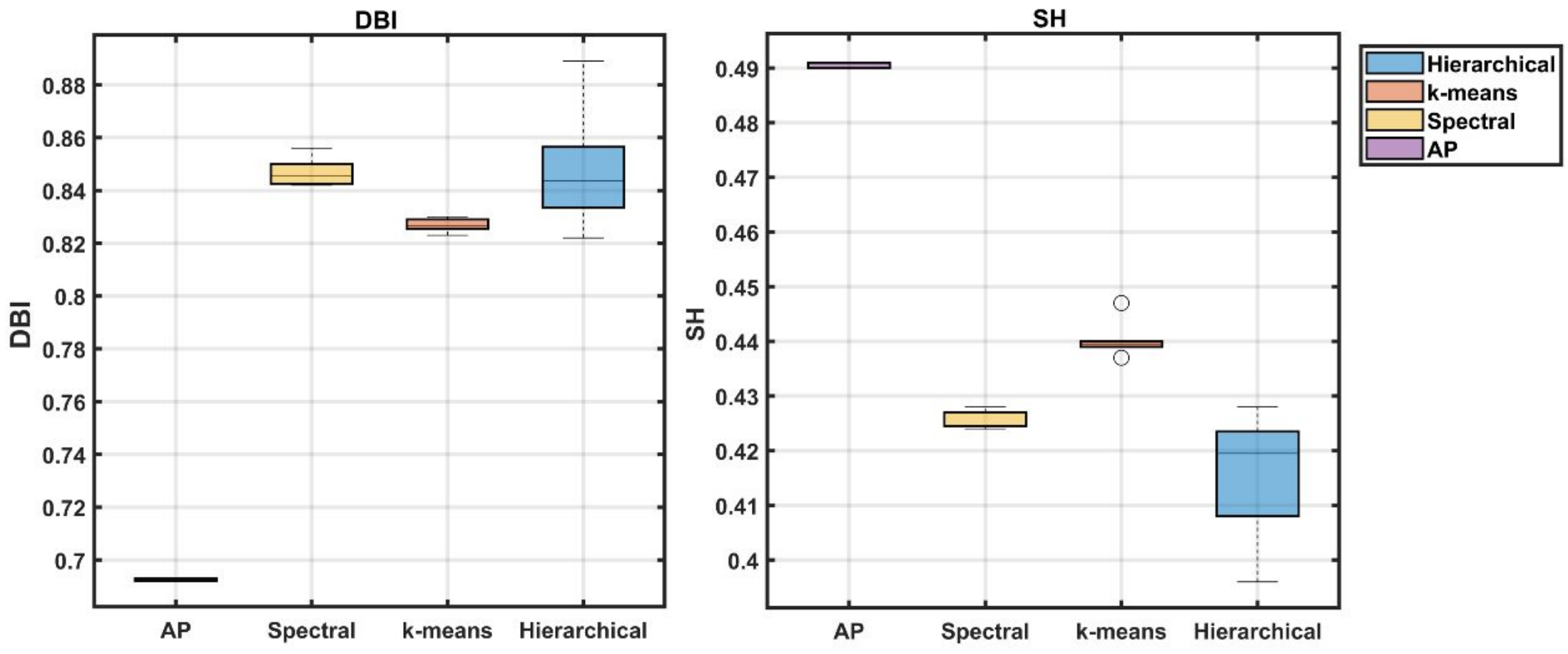

Figure S3 Boxplots showing the distributions of the Davies-Bouldin index (DBI, left) and silhouette coefficient (SH, right) for Affinity Propagation (AP), spectral clustering, k-means and hierarchical clustering under different noise conditions (noise levels ranging from 0.5 to 4). AP exhibited the most stable performance across all noise levels, with consistently low DBI values, persistently high SH values and minimal fluctuations, demonstrating strong robustness to noise. In contrast, spectral clustering performed the weakest, with a marked decline in performance under high noise conditions. K-means and hierarchical clustering showed moderate sensitivity to noise, with substantially larger performance fluctuations than AP.

With the validated algorithm and parameters, we successfully obtained a stable and reproducible three-subregion parcellation of the primary visual cortex (V1). Using the improved Affinity Propagation (AP) clustering algorithm based on the structure-function coupling (SFC) matrix, we partitioned V1 into three spatially contiguous subregions, establishing the optimal scheme for dividing V1 into three distinct zones. This parcellation reveals a complex functional organisation within V1, where each subregion likely undertakes distinct visual information processing tasks, reflecting both the diversity and specialisation of the V1 region. More importantly, this division may correspond to different pathways of visual information processing (e.g. the functional division of the dorsal and ventral streams), suggesting that V1 subregions are specialised in processing specific visual features such as motion, shape and colour.

The scheme divides V1 into three subregions: cranial (Cra-V1), dorsal (Dor-V1) and caudal (Cau-V1) (Figure S4). We provide detailed annotations for the clustering results, with subregions labelled and colour-coded as follows: Cra-V1 (green), Dor-V1 (red) and Cau-V1 (blue). This fine-grained subdivision of V1 not only deepens our understanding of the functional organisation of the visual cortex but also offers a new perspective for investigating the mechanisms of visual information processing and the neural basis of related visual impairments.

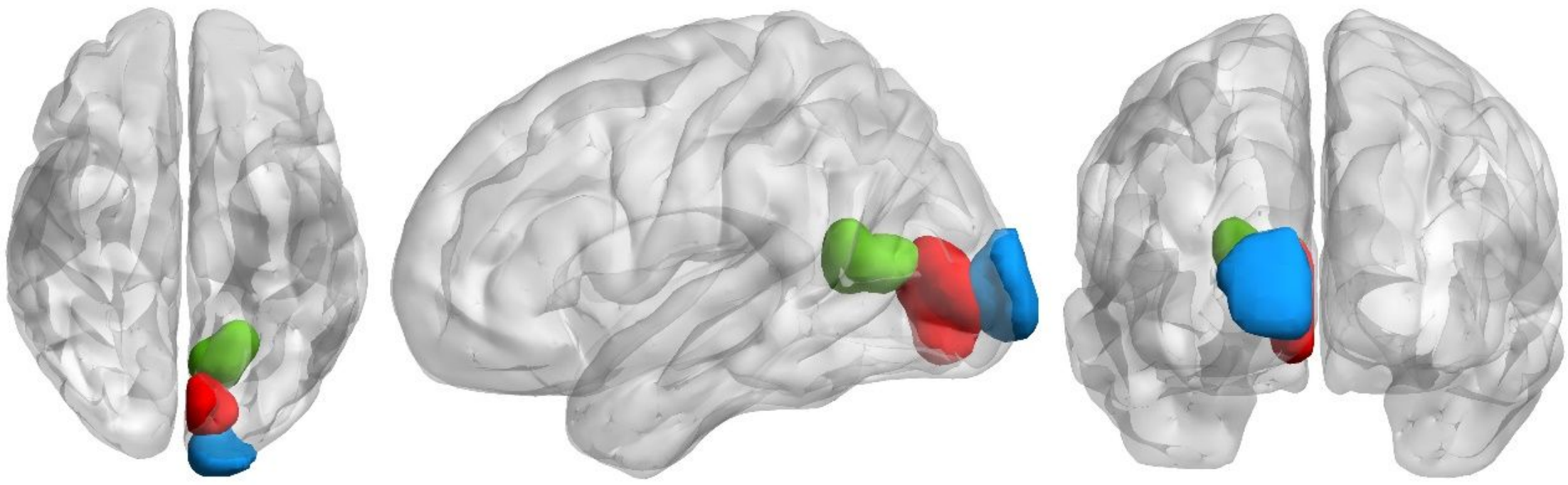

Figure S4 shows three spatially contiguous functional subregions: the cranial (Cra-V1), dorsal (Dor-V1) and caudal (Cau-V1) regions (Figure S3). Cra-V1 is indicated in green, Dor-V1 in red and Cau-V1 in blue. This subregion not only reflects the anatomical gradient within V1 but also likely corresponds to its early functional specialisation. Specifically, the dorsally located subregion (Dor-V1) is spatially adjacent to the input sources of the dorsal visual pathway (the "where" pathway, responsible for spatial localisation and motion processing), while the caudal subregion (Cau-V1) is closer to the projection areas of the ventral visual pathway (the "what" pathway, responsible for object recognition). The cranial subregion (Cra-V1) may play a role in the integration of early visual features. This tripartite division based on connectivity patterns provides a novel spatial framework for understanding how V1 serves as the functional origin of distinct higher-order visual pathways.

To further evaluate the contribution of multimodal information, we compared the improved AP algorithm using the SFC matrix with the same algorithm using FC alone. The SFC-based solution achieved a higher silhouette coefficient (0.5197 vs 0.4716) and a lower Davies-Bouldin index (0.6936 vs 0.7701) than the FC-based solution, indicating more compact and better-separated clusters (Figure S5).

Functional homogeneity (FH) analysis further showed that the SFC matrix yielded higher FH values across the three subregions (subregion 1: 0.748 ± 0.021; subregion 2: 0.745 ± 0.018; subregion 3: 0.753 ± 0.015) than the FC matrix alone (0.730 ± 0.023, 0.718 ± 0.019, 0.660 ± 0.027). Because no inferential test is reported for this comparison, we describe these differences as higher rather than statistically significant. The SC-based representation showed the highest FH values (0.773-0.870), suggesting that anatomical connectivity contributed strongly to the spatial coherence of the SFC-derived parcellation (Figure S6).

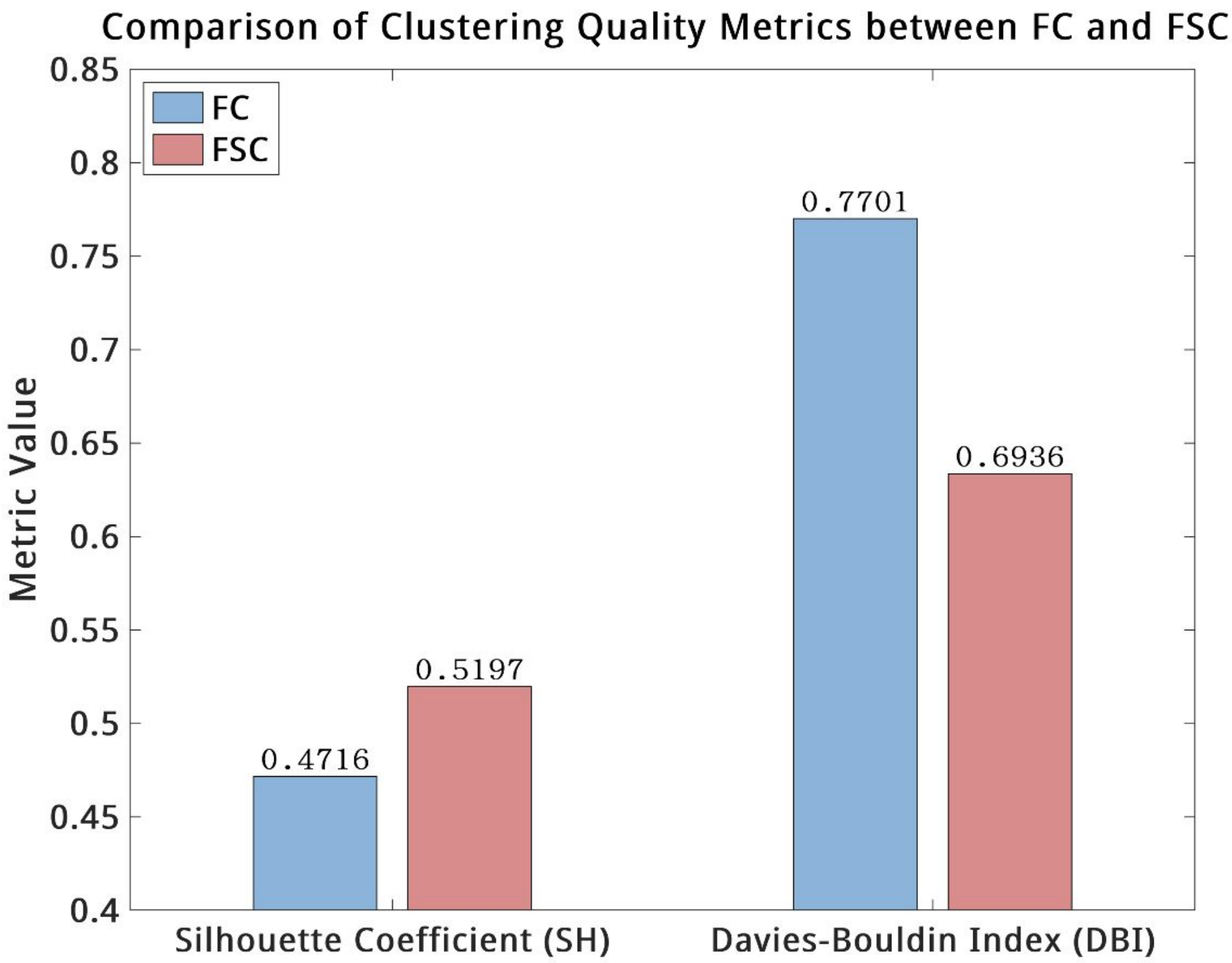

Figure S5 Bar chart comparing the silhouette coefficient (SH) and Davies-Bouldin index (DBI) derived from the functional connectivity (FC) matrix and the structure-function coupling (SFC) matrix proposed in this study. Higher SH values and lower DBI values indicate superior clustering performance. Compared with the FC representation, the SFC representation yielded a higher SH value (0.5197 vs 0.4716) and a lower DBI value (0.6936 vs 0.7701), demonstrating that SFC-based representation provides more compact and better-separated clustering results for V1 subregion.

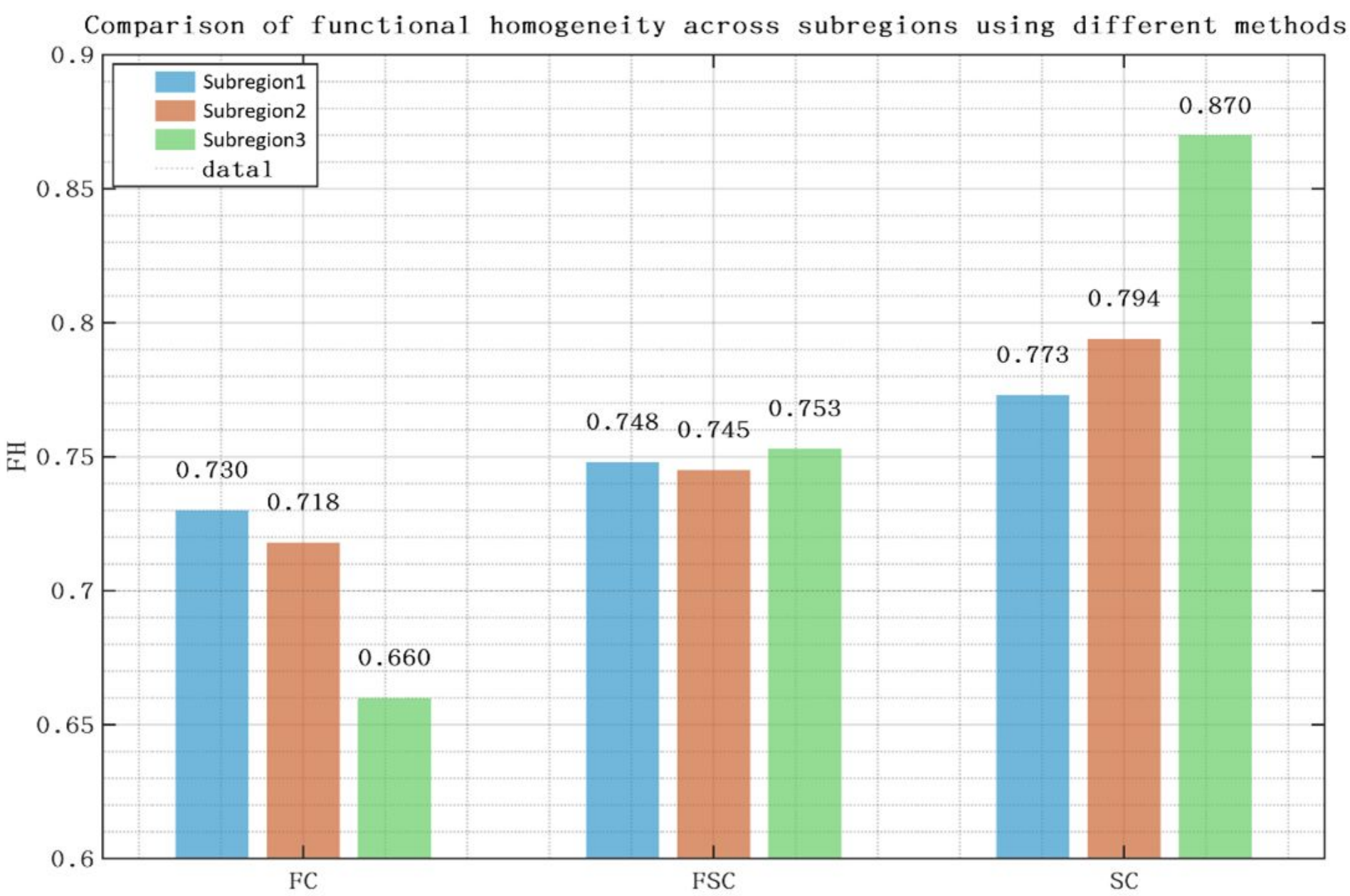

Figure S6 Comparison of functional homogeneity across V1 subregions based on different connectivity representations. Bar chart showing

the functional integration index (FH) for the three subregions (Subregion 1, 2, 3) calculated from the functional connectivity (FC) matrix, the structure-function coupling (SFC) matrix proposed in this study and the structural connectivity (SC) matrix. Across all methods, the SC-based calculation yielded the highest FH values in all subregions, indicating the strongest intra-subregion functional homogeneity. The SFC representation proposed here also achieved higher homogeneity than the conventional FC method.

Finally, to translate theoretical findings into clinical value, we applied the above parcellation scheme to an empirical analysis of autism spectrum disorder (ASD). For group difference and clinical correlation analyses, we used data from the NYU site. Based on the optimal clustering solution determined by the silhouette coefficient (SH), i.e., the three-parcellation of the primary visual cortex (V1), we extracted connectivity features from the left and right hemispheres of both the ASD and typically developing (TD) groups at NYU and performed comparative analyses (see Figure S7 for details). After false discovery rate (FDR) correction ($p < 0.05$), we found that in the ASD group, amplitude of low-frequency fluctuation (ALFF) was significantly lower than in the TD group in the left-hemisphere dorsal V1 (Dor-V1), cranial V1 (Cra-V1) and caudal V1 (Cau-V1), indicating weakened spontaneous neural activity in these regions, potentially reflecting reduced functional connectivity and impaired visual information integration in ASD. Conversely, degree centrality (DC) was significantly higher in the right-hemisphere Cra-V1 and Dor-V1 in ASD than in TD, suggesting enhanced functional connectivity, possibly related to visual hypersensitivity and excessive information processing. Beyond significance testing (p values), we calculated effect sizes (Cohen's d) to quantify the magnitude of group differences: Cohen's d represents the standardised difference between two group means, with conventions of $d = 0.2$ (small), 0.5 (medium), and ≥0.8 (large). Results showed: for the left hemisphere, ALFF in Cau-V1 ($d = 0.767$, medium effect) and Dor-V1 ($d = 0.604$, medium effect), and DC in Cra-V1 ($d = 0.608$, medium effect) and Dor-V1 ($d = 0.557$, medium effect); for the right hemisphere, ALFF in Dor-V1 ($d = 0.762$, medium effect) and Cau-V1 ($d = 0.555$, medium effect), and DC in Cra-V1 ($d = 0.557$, medium effect) and Dor-V1 ($d = 0.608$, medium effect). These intergroup differences were not only statistically significant but also mostly of medium effect size ($d = 0.555$-0.767), indicating clinically meaningful distinctions in spontaneous neural activity and functional connectivity patterns between ASD and TD within the V1 subregions. Specifically, left-hemisphere ALFF reductions suggest diminished spontaneous activity may affect visual motion processing or spatial integration, while right-hemisphere DC increases (medium effects, particularly in Dor-V1 and Cra-V1) may reflect excessive integration or compensatory enhancement of visual information in the right hemisphere.

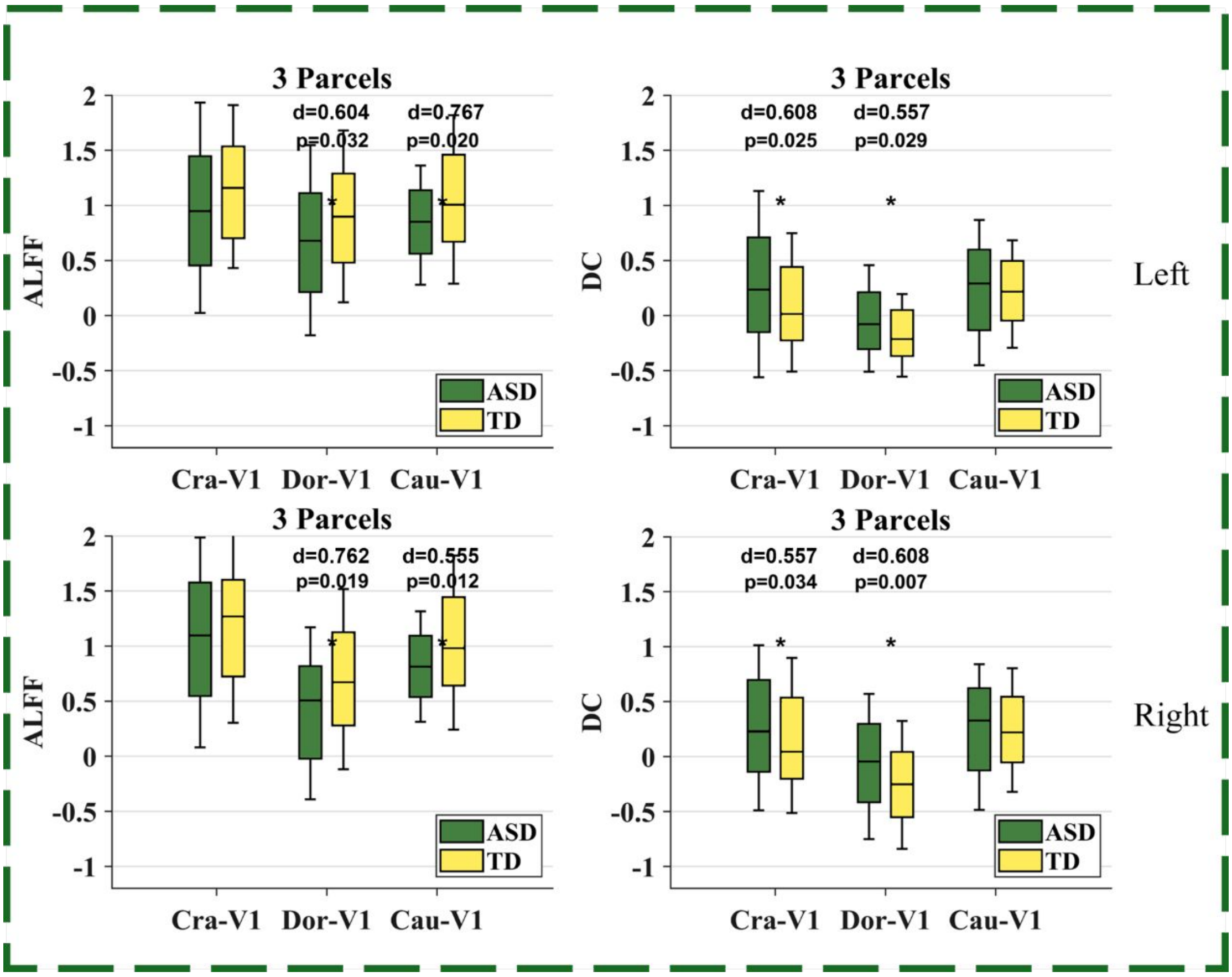

Figure S7. Based on the three-cluster results of V1, we conducted inter-group difference comparisons between the ASD group and the TD group. In Figure (a), we compared the ALFF feature, while in Figure (b), we compared the DC feature. It is important to note that the abbreviations in the figure are defined as follows: Cra represents the cranial region, Dor represents the dorsal region and Cau represents the caudal region. In the figure, data points marked with an asterisk (*) indicate a p value of less than 0.05 after FDR correction. The symbol d denotes the effect size measure (Cohen's d).

In addition, we applied the optimal clustering results to clinical data from the NYU site and used multiple clinical assessment tools (including the Autism Diagnostic Interview-Revised [ADI-R], Autism Diagnostic Observation Schedule [ADOS], Social Responsiveness Scale [SRS] and Vineland Adaptive Behavior Scales [VINELAND]) to evaluate associations between functional connectivity abnormalities in the primary visual cortex (V1) and core symptoms of ASD. Figure S8-A and S8-B show correlations between different V1 subregions and clinical scores. After FDR correction, significant negative correlations were observed in the left hemisphere between interhemispheric functional connectivity (VMHC) of the dorsal subregion (Dor-V1) and the total score of the ADI-R social domain, total ADOS score and ADOS social domain score. In the right hemisphere, ALFF in Dor-V1 showed a significant negative correlation with the total onset-age score of ADI-R; fractional amplitude of low-frequency fluctuation (fALFF) in this subregion was significantly negatively correlated with the total ADI-R social domain score, total ADOS score and ADOS social domain score; furthermore, VMHC in Dor-V1 was significantly negatively correlated with the ADOS restricted and repetitive behaviour subscale score. In the region-of-interest-to-

region-of-interest (ROI-to-ROI) functional connectivity (FC) group difference analysis, four statistically significant ROI-to-ROI functional connectivity differences were identified. These corrected p values indicate statistically significant ROI-to-ROI functional connectivity differences, supporting the robustness of the observed group effects. These results demonstrate significant negative correlations between functional connectivity of V1, particularly the dorsal subregion, and core symptoms of ASD: specifically, interhemispheric connectivity and low-frequency oscillatory activity in Dor-V1 are significantly associated with social deficits and restricted/repetitive behaviours in individuals with ASD. This suggests that functional abnormalities in dorsal V1 may play a key role in the pathophysiology of ASD, potentially disrupting social cognitive function by affecting visual information processing. Our findings also highlight the importance of the primary visual cortex, especially V1, in ASD and suggest this region as a potential target for future ASD diagnosis and intervention.

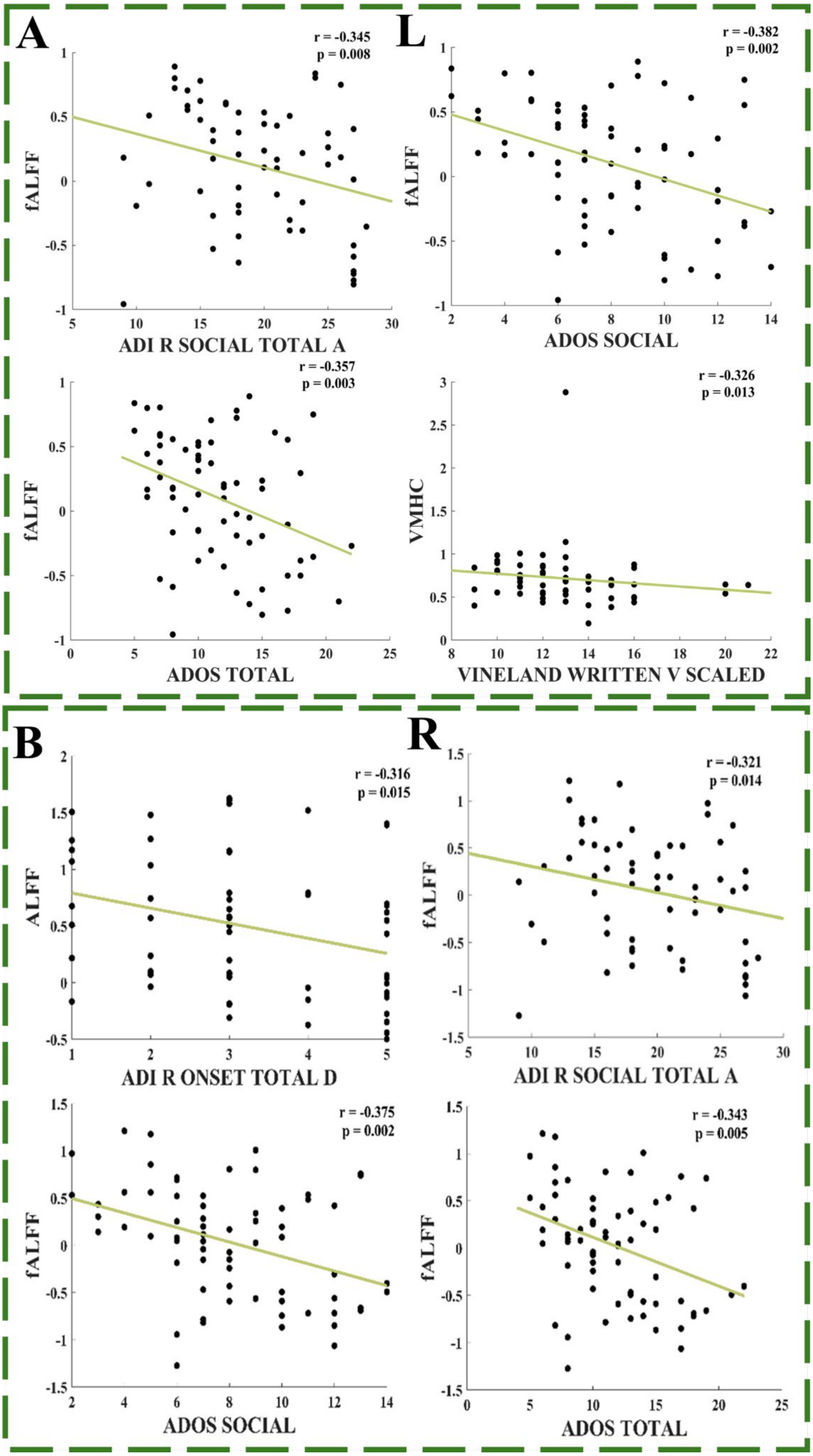
A
r = -0.345
p = 0.008
fALFF
ADI R SOCIAL TOTAL A
r = -0.357
p = 0.003
fALFF
ADOS TOTAL
L
r = -0.382
p = 0.002
fALFF
ADOS SOCIAL
r = -0.326
p = 0.013
VMHC
VINELAND WRITTEN V SCALED
B
r = -0.316
p = 0.015
ALFF
ADI R ONSET TOTAL D
r = -0.375
p = 0.002
fALFF
ADOS SOCIAL
R
r = -0.321
p = 0.014
fALFF
ADI R SOCIAL TOTAL A
r = -0.343
p = 0.005
fALFF
ADOS TOTAL

Figure S8. **(A)** Scatter plots of clinical correlations for the second subregion of the left visual cortex. In this subregion, three clinical scores passed FDR correction for the fractional amplitude of low-frequency fluctuation (fALFF) feature, and one clinical score passed FDR correction for the voxel-mirrored homotopic connectivity (VMHC) feature. (B) Scatter plots of clinical correlations for the second subregion of the right visual cortex. In this subregion, one clinical score passed FDR correction for the VMHC feature, one clinical score passed FDR correction for the amplitude of low-frequency fluctuation (ALFF) feature, and three clinical scores passed FDR correction for the fALFF feature.
